# The ruminal bacterial community in lactating dairy cows has limited variation on a day-to-day basis

**DOI:** 10.1186/s40104-019-0375-0

**Published:** 2019-08-19

**Authors:** Joseph H. Skarlupka, Maria E. Kamenetsky, Kelsea A. Jewell, Garret Suen

**Affiliations:** 1 Department of Bacteriology, University of Wisconsin-Madison, 5159 MSB, 1550 Linden Drive, Madison, WI 53706 USA; 2 Department of Population Health Sciences, University of Wisconsin-Madison, Madison, 53726 USA; 3 College of Agricultural and Life Sciences Statistical Consulting Lab, University of Wisconsin-Madison, Madison, 53706 USA; 4 Department of Biology, Centralia College, Centralia, WA 98531 USA

**Keywords:** Bacterial community, Next-generation sequencing, Rumen microbiota

## Abstract

Dairy cows rely on a complex ruminal microbiota to digest their host-indigestible feed. Our ability to characterize this microbiota has advanced significantly due to developments in next-generation sequencing. However, efforts to sample the rumen, which typically involves removing digesta directly from the rumen via a cannula, intubation, or rumenocentesis, is costly and labor intensive. As a result, the majority of studies characterizing the rumen microbiota are conducted on samples collected at a single time point. Currently, it is unknown whether there is significant day-to-day variation in the rumen microbiota, a factor that could strongly influence conclusion drawn from studies that sample at a single time point. To address this, we examined day-to-day changes in the ruminal microbiota of lactating dairy cows using next-generation sequencing to determine if single-day sampling is representative of sampling across 3 consecutive days. We sequenced single-day solid and liquid fractions of ruminal digesta collected over 3 consecutive days from 12 cannulated dairy cows during the early, middle, and late stages of a single lactation cycle using the V4 region of the bacterial 16S rRNA gene. We then generated 97% similarity operational taxonomic units (OTUs) from these sequences and showed that any of the individual samples from a given 3-day sampling period is equivalent to the mean OTUs determined from the combined 3-d data set. This finding was consistent for both solid and liquid fractions of the rumen, and we thus conclude that there is limited day-to-day variability in the rumen microbiota.

**Electronic supplementary material:**

The online version of this article (10.1186/s40104-019-0375-0) contains supplementary material, which is available to authorized users.

## Introduction

The ruminal microbial community plays a significant role in the health and productivity of foregut fermenters [1]. These microbes are necessary to ferment plant matter into compounds such as volatile fatty acids that can be metabolized by the host. Much like the dynamic intestinal microbial communities of other animals, the rumen microbiota can change over time with respect to composition due to numerous variables including age, diet, pregnancy, and illness [2]. The relationship between the composition of the rumen microbial community and its contributions to the host has become a focus in dairy science, especially with the introduction and development of next-generation sequencing technologies that enable rapid investigation of large-scale community membership. For example, Jami et al. [3] and Jewell et al. [4] used next-generation sequencing to demonstrate strong correlations between the composition of the ruminal bacterial community and both milk yield and milk production efficiency in dairy cows, respectively.

Numerous methods for sampling the rumen have been employed, including buccal swabs, bolus samples, and digesta samples directly from the rumen. In terms of non-invasive proxies of rumen microbial communities, buccal and bolus samples are much more representative of the rumen community than fecal samples because cows regurgitate the microbe-laden digesta and chew it before swallowing it again, leaving some of the microbes behind in the mouth [5, 6]. However, sampling of the total rumen digesta, albeit more difficult, is by far the most direct method for collecting the entire rumen microbial community, and can be accomplished by stomach tubing, rumenocentesis, or through a cannula.

While it has been shown that the ruminal bacterial community structure changes over long periods of time (e.g., a typical 305-day lactation period [4]), almost nothing is known about how this community changes on a day-to-day basis. Using automated ribosomal intergenic spacer analysis, Weimer et al. [7] showed day-to-day consistency in the rumen microbiota, but this approach has limited resolution, relative to more advanced approaches such as next-generation sequencing. As a result, while some studies of the rumen microbiota collect samples over multiple days to average the microbial community and account for any potential day-to-day variability [6, 7], the majority of reports rely on single day sampling and assume that there is no variability in the short term. This presents a critical issue in the interpretation of these data if there is significant variation in the rumen microbiota on a day-to-day basis and may bring into question the conclusions drawn from those studies. However, if little to no variation is observed in the rumen microbiota on a day-to-day basis, this would not only validate previous work, but also allow researchers to continue single-day sampling and decrease the amount of time and labor required to accurately characterize the rumen microbial community.

The goal of this study was to determine if the rumen bacterial community changes significantly on a day-to-day basis. We hypothesized that there is limited variability in the rumen microbiota on a day-to-day basis, and we tested this by sequencing ruminal samples collected from 12 lactating Holstein dairy cows over 3 days during three different periods of a single lactation cycle.

## Materials and methods

### Sample collection

The samples used in this study were collected as part of a previous report that examined the correlation between feed efficiency in lactating dairy cows and their ruminal bacterial communities [4]. All samples were collected according to Research Animal Resource Center (RARC) protocol A01104, approved by the University of Wisconsin-Madison College of Agriculture and Life Sciences Institutional Animal Care and Use Committee. In brief, phase-separated rumen samples from 12 lactating Holstein dairy cows were collected via cannula just prior to once-daily feeding for 3 consecutive days within 3 periods of a lactation cycle (early, middle, late). All animals in the study were approximately 2 years old at the beginning of the study. Sampling was done across the full first lactation cycle, for a total of 216 samples. Detailed sampling methods and additional information about the cows used in this study can be found in [4].

### DNA extraction, PCR, and library preparation

DNA extraction, using a mechanical cellular disruption method and hot/cold phenol, was performed similar to a method detailed by Henderson et al. [8] that generates high yields of DNA representative of the ruminal community. There was not enough DNA for amplification of a single sample (Sample L82.1E3), reducing the total number of samples used in this study to 215. Sample DNA was quantified using a Qubit fluorometer reagents (Invitrogen, Waltham, MA) and a Synergy 2 microplate reader (BioTek, Winooski, VT, USA). The V4 region of the 16S rRNA gene was amplified via polymerase chain reaction (PCR) using a universal bacterial primer (F- GTGCCAGCMGCCGCGGTAA; R- GGACTACHVGGGTWTCTAAT), as described by Kozich et al. [9]. These primers also included adapters suitable for sequencing using the Illumina technology (F- AATGATACGGCGACCACCGAGATCTACAC; R- CAAGCAGAAGACGGCATACGAGAT) and further included unique barcodes to facilitate multiplexing: the forward primers had 16 unique 8-bp barcodes, and the reverse primers had 24 unique 8-bp barcodes. A total of 25–50 ng of DNA and 0.2 μmol/L of primer were combined in a 25-μL reaction with 2× KAPA HiFi HotStart ReadyMix (KAPA Biosystems, Wilmington, MA). The reactions were run on a Bio-Rad S1000 thermocycler (Bio-Rad Laboratories, Hercules, CA, USA) with the following conditions: 95 °C for 3 min, 25 cycles of 95° for 30 s, 55 °C for 30 s, and 72 °C for 30 s, followed by a final extension at 72 °C for 5 min.

PCR products were quantified on a 1% (w/v) low-melt agarose gel using AquaPor low-melt agarose (National Diagnositcs, Atlanta, GA) using SYBRSafe DNA gel stain (Invitrogen, Waltham, CA), and bands at ~ 380 bp indicated successful amplification. These bands were excised, extracted, and cleaned using a Zymoclean Gel DNA Recovery Kit (Zymo Research, Irving, CA). A no-template negative control was included with each set of PCRs and if a band was present in the negative control, all samples in that set were redone starting at PCR set-up and amplification. Negative controls for which no band was present had the approximate location of the amplicon (~ 380 bp) excised and sequenced as further confirmation that no contamination was present. Gel-extracted DNA was then quantified using on a Qubit fluorometer and a 96-well plate spectrophotometer, and a library was created using a 4 nmol/L equimolar pool of all PCR products. This library was then sequenced on an Illumina MiSeq following standard Illumina sequencing protocols using a MiSeq v2 2 × 250 sequencing kit at 10 pmol/L and with a 10% PhiX control. All sequences associated with this study were deposited into the National Center for Biotechnological Information’s Short Read Archive and is available under BioProject Accession SRP150748.

### Sequence analysis

The resulting fastq files from the sequencer were subjected to cleanup and analysis using mothur v1.39.0 [10]. Sequences were screened (maxambig = 0, maxhomop = 8, minlength = 200, maxlength = 500), and identical sequences were grouped using *unique.seqs*. The sequences were then aligned (*align.seqs*) to the SILVA 16S rRNA gene reference alignment database (Release 132) [11] and again screened for sequences aligned to our region of interest (*screen.seqs*, start = 13,862, end = 23,444). Sequences were then filtered (*filter.seqs*) and identical sequences grouped together (*unique.seqs*). Highly similar sequences were grouped using *pre.cluster* (diffs = 2), with *chimera.uchime* and *remove.seqs* used to detect and remove chimeras, respectively. Sequences were classified using the SILVA database and those that were non-bacterial (unknown at the Kingdom level, Archaea, Eukaryota, cyanobacteria, and mitochondria) were removed.

Those sequences that appeared only once in the dataset were removed (*split.abund*) so as to minimize bias due to sequencing error, and the uncorrected pairwise distances between the sequences was calculated (*dist.seqs*). Sequences were then assigned to operational taxonomic units (OTUs) with *cluster.split* (method = opti, cutoff = 0.03) at a 97% sequence similarity. Good’s index [12] was used to determine sample coverage, and the taxonomy of the OTUs were determined using the GreenGenes database (August 2013 release) [13]. Finally, the samples were normalized to the lowest number of sequences found in all samples, which was 9100 sequences. A 0.1% total abundance cutoff was applied to the normalized dataset using *filter.shared* (minpercent = 0.001) and a total community structure analysis was performed using non-metric multidimensional scaling (NMDS) of the Bray-Curtis dissimilarity index [14].

### Statistical analysis

All statistical analyses were performed in R version 3.4.4 [15]. For our analysis, we first constructed a table of counts of OTUs across all cows containing both the solid and liquid samples (Additional file 1: Table S1) and then compared 1 day and combined 3-day samplings by creating three tables of OTU abundances from this dataset: one that was a random day from every three-day period, a second that used the means of the OTU abundances from all 3 d, and a third that used the median of the OTU abundances across all 3 d. To address normality, we performed a natural log transformation of the three OTU tables.

To determine if 1 day and 3-day sampling were equivalent, we used the two one-sided test (TOST) of equivalency for paired data using the “equivalence” R package [16]. As opposed to a classical t-test, the TOST sets the null hypothesis as the two mean values not being equivalent and sets the alternative to be equivalent. Therefore, when there is strong evidence to reject the null hypothesis, it is done in favor of the alternative hypothesis that the two means are equivalent. We set the equivalency margin ε = 0.25 and used the paired TOST on the random day vs. the mean OTU datasets and the random day vs. the median OTU datasets. We performed the same tests two more times using tables of OTUs for all animals, but split into solids and liquids.

To address distributional concerns and to minimize assumptions, we performed the robust TOST following Yuen’s robust test [17, 18], which makes no assumptions of normality and is more robust to outliers and long-tailed distributions. An adjusted mean, where a percentage of the largest and smallest observations are removed, or trimmed, is used. This has been shown to result in a minimal loss of power efficiency under exact normality, but with appreciable gains for longer-tailed distributions [17]. All R code for the creation of the OTU tables used in the equivalency tests and the tests themselves can be found in Additional file 2: Supplementary Methods.

## Results

### Microbiota sequencing results

In total, we generated 12,530,956 raw sequences, of which 8,212,441 were high-quality reads that passed through the cleanup process. The coverage for all samples was deemed adequate, at > 0.95 Good’s index for each sample. The number of sequences in each sample ranged from 10,397 - 196,077, with a mean of 38,198 ± SD 23,005 and a median of 35,750 ± 22,928 sequences. When separated by phase, the liquid fraction ranged between 10,397–196,077 sequences, while the solid fraction ranged from 12,249–89,318 sequences. With all samples combined, 19,217 unique OTUs were identified at 97% sequence similarity. The number of OTUs within samples ranged between 603 and 2358 and 746–2018 for liquid and solid samples, respectively. The mean and median number of OTUs observed in each individual sample was 1370 ± 360 and 1367 ± 360, respectively.

### Single-day microbiota samples are equivalent to 3-day pooled samples

We tested our samples using the TOST and RTOST and found that for all animals with solid and liquid samples in the same OTU table, both comparisons of the OTU abundances from a random single-day sample against the mean or median of the associated 3-day samples were significant (*P* <  0.0001, Table 1), providing evidence to reject the null hypothesis. We found similar results for the comparisons of only the solid and only the liquid samples (*P* <  0.0001, Table 1). This indicates that the random vs. mean and random vs. median datasets are rejected to be not equivalent at the 5% level and favors the alternative hypothesis of them being equivalent. Further analysis using the robust paired TOST on the same comparisons, with a trim level of 20% and equivalency margin of 0.25, resulted in significance (*P* <  0.0001, Table 1) for both comparisons, further confirming strong evidence to reject the null hypothesis that the two are not equivalent at the 5% level.

**Table 1:** *P* values from TOST and RTOST analyses of the solid, liquid, and combined OTU tables. A random day was taken from the OTU table for each period of lactation for each cow and compared against the mean and the median of the OTU count for that period of the same cow

Sample type	Random vs Mean	Random vs Median
TOST *P* values		
Solids	< 0.0001	< 0.0001
Liquids	< 0.0001	< 0.0001
Combined	< 0.0001	< 0.0001
RTOST *P* values		
Solids	< 0.0001	< 0.0001
Liquids	< 0.0001	< 0.0001
Combined	< 0.0001	< 0.0001

### Non-metric multidimensional scaling analysis show little day-to-day variation

**Fig. 1:**
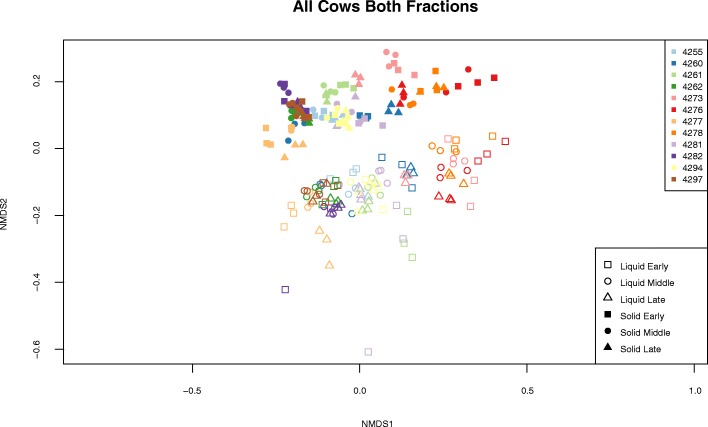
Non-metric multidimensional scaling plot of the ruminal solid and liquid phase microbiota from 12 lactating dairy cows sampled during early, middle, and late periods of their first lactation cycle. Distances were calculated using the Bray-Curtis dissimilarity index. Points are plotted based on individual cow, period of lactation, and type of sample

We also examined the differences between both the samples within a group and across groups by plotting each day of sampling for all cows, based on lactation period, using non-metric multidimensional scaling (NMDS) of the Bray-Curtis dissimilarity index. Samples collected from the same 3-day period tended to form clear, distinct, groups for each cow across lactation stages, and the tight clustering of each of these samples suggests that these communities do not vary on a day-to-day basis (Fig. 1). Analysis of liquid and solid fractions for all cows showed a clear separation based on phase, and tight clustering within each phase according to cow and lactation period (Additional file 3: Figures S1 and S2).

## Discussion

In this study we show that, while there are changes in the ruminal communities over time, the changes between successive days is not as large as the changes present between individuals and rumen phase. This is evidenced by our paired TOST and robust paired TOST equivalency tests, which indicate that the OTUs from a randomly-selected sampling day is equivalent to the mean and median of each pooled 3-day period. Our Bray-Curtis NMDS plots further support our findings by demonstrating tight clustering within periods. It should be noted that our sampling of the rumen occurred prior to the once-daily feeding period for each cow, and it is possible that samples collected during or after feeding may vary relative to before-feeding samples due to the introduction of extraneous microbes from feed and the influx of nutrients enriching for particular bacteria. Nonetheless, our work demonstrates that the ruminal microbiota obtained from a single-day sampling of the rumen is equivalent to samples collected over multiple successive days within the same period, and that there is little day-to-day variation. Our findings both confirm and validate previous studies that rely upon single-day sampling of the rumen before feeding and we suggest that this approach can be used for future studies of the rumen microbiota.

## Conclusions

We conclude that there is little day-to-day variation in the ruminal bacterial community structure of lactating Holstein dairy cows, and that this property is consistent across multiple time points during a lactation cycle. Our work validates previous studies that document the ruminal microbiota using single-day sampling and further enables future studies to utilize single-day sampling without concern that day-to-day variation in the ruminal microbiota might complicate the resulting conclusions.

## Additional files

Additional file 1: **Table S1.** Taxonomy and Sequence Counts of all OTUs with >0.1% Sequence Count Abundance found in all animals at all time points. (XLSX 158 kb)

Additional file 2: R Code for the TOST and RTOST tests and NMDS Plotting. (PDF 1017 kb)

Additional file 3: **Figures S1** and **S2**. Bray-Curtis NMDS Plots of the Ruminal Solid and Liquid Fractions. (DOCX 28 kb)

## Data Availability

All sequences associated with this study have been deposited into the National Center for Biotechnological Information’s Short Read Archive and is available under BioProject Accession SRP150748.

## Abbreviations

NMDS: Non-Metric Multidimensional Scaling
OTU: Operational Taxonomic Unit
RTOST: Robust Two One-Sided Test
TOST: Two One-Sided Test
