# Supplementary material for: The ruminal bacterial community in lactating dairy cows has limited variation on a day-to-day basis
Source: J Anim Sci Biotechnol. 2019 Aug 19;10:66. doi: 10.1186/s40104-019-0375-0 (PMC6698983; doi:10.1186/s40104-019-0375-0)
Supplement: Supplementary file 2 — R Code for the TOST and RTOST tests and NMDS Plotting. (PDF 1017 kb) [file 40104_2019_375_MOESM2_ESM.pdf]

Paired TOST & NMDS

Maria Kamenetsky & Joseph Skarlupka

April 5, 2018

Paired TOST Analysis

First, we read in and prepare the data.

```
#read in data
cowsAll <- read.table("FirstYearShared.txt", header = TRUE)
```

```
#Load libraries
library(equivalence)
library(dplyr)
```

```
#Splitting data into mean OTUs, median OTUs, and random day OTU
cowsAll$ID <- substr(cowsAll$Group,0,3)
cowsAll$lactationround <- substr(cowsAll$Group,5,5)
cowsAll$period <- substr(cowsAll$Group,6,6)
cowsAll$rep <- substr(cowsAll$Group,7,7)
cowsAll$cowperiod <- paste0(cowsAll$ID, cowsAll$period)

#all mean
collapsedAll_mean <- aggregate(cowsAll[,2:160], list(cowsAll$cowperiod), mean)
collapsedAll_mean <- collapsedAll_mean[,-1]
collapsedAll_mean <- as.matrix(collapsedAll_mean)


#all median
collapsedAll_median <- aggregate(cowsAll[,2:160], list(cowsAll$cowperiod), median)
collapsedAll_median <- collapsedAll_median[,-1]
collapsedAll_median <- as.matrix(collapsedAll_median)


#all random
set.seed(422018)
collapsedAll_random <- cowsAll[,c(2:160,165)] %>% group_by(cowperiod) %>% sample_n(1)
collapsedAll_random <- sapply(collapsedAll_random[,-160], as.numeric)
collapsedAll_random <-  as.matrix(collapsedAll_random)
```

Next, we explore our data:

```
#Summary stats
summary(as.vector(collapsedAll_random))
```

```
##    Min. 1st Qu.  Median    Mean 3rd Qu.    Max. 
##    0.00    6.00   16.00   56.86   35.00 3760.00
```

Although the third quartile is at 35, we see that the maximum is over 3000. We want to check this observations:

```
#RANDOMLY Selected Day
length(which(collapsedAll_random>3000,arr.ind = TRUE)) #there are 52 observations > 3000
```

```
## [1] 52
```

```
#Exploring these further
View(collapsedAll_random[,c(1,159)])
#it appears this large counts are in the first OTU column and the rare OTU column
##visual checks and exploration
jj <- scale(as.vector(collapsedAll_random))
qqnorm(jj, main = "QQ-Plot for Randomly Selected Day")
abline(0, 1, col="red")
```

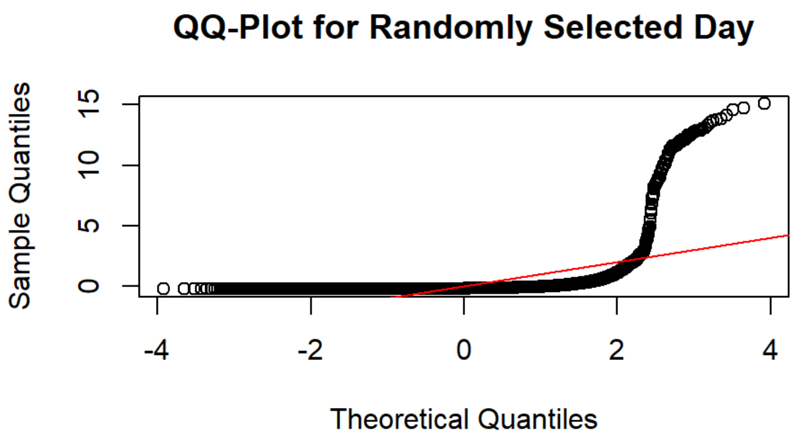


```
plot(density(as.vector(collapsedAll_random)), main="Density Plot for Randomly Selected Day")
```

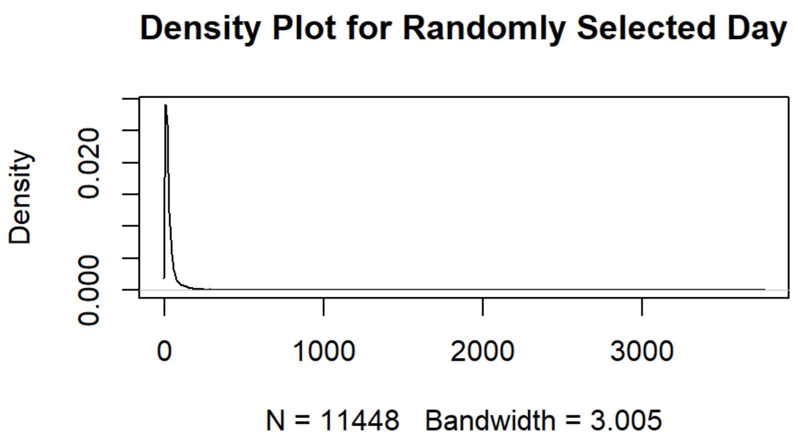


```
#check MEAN
summary(as.vector(collapsedAll_mean))
```

```
##     Min.  1st Qu.   Median     Mean  3rd Qu.     Max.
##    0.000    7.333   16.333   56.902   35.667 3686.667
```

```
length(which(collapsedAll_mean>3000,arr.ind = TRUE)) #there are 50 observations > 3000
```

```
## [1] 50
```

```
View(collapsedAll_mean[,c(1,159)])
##visual check and exploration
jj <- scale(as.vector(collapsedAll_mean))
qqnorm(jj, main = "QQ-Plot for Mean")
abline(0, 1, col="red")
```

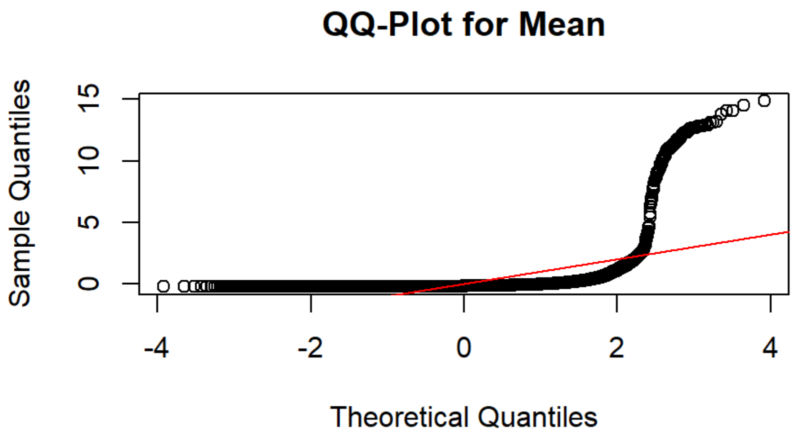


```
plot(density(as.vector(collapsedAll_random)), main="Density Plot for Mean")
```

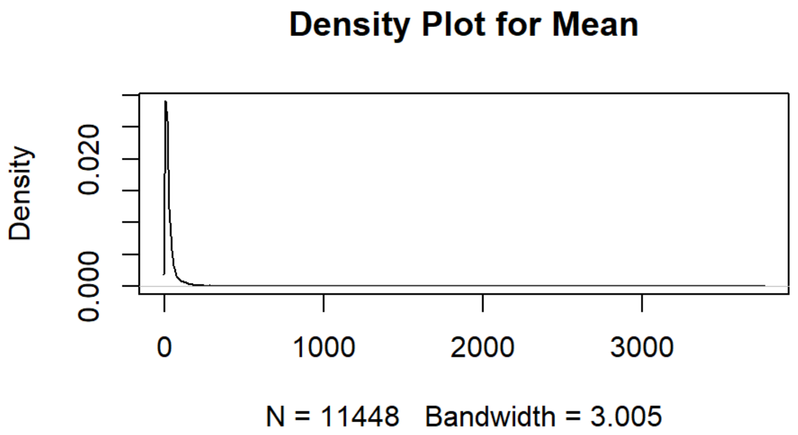


```
#check MEDIAN
summary(as.vector(collapsedAll_median))
```

```
##    Min. 1st Qu.  Median    Mean 3rd Qu.    Max.
##    0.00    7.00   16.00   56.35   35.00 3673.00
```

```
length(which(collapsedAll_median>3000,arr.ind = TRUE))#there are 52 observations > 3000
```

```
## [1] 52
```

```
View(collapsedAll_median[,c(1,159)])
##visual check
jj <- scale(as.vector(collapsedAll_median))
qqnorm(jj)
abline(0, 1, col="red", main = "QQ-Plot for Median")
```

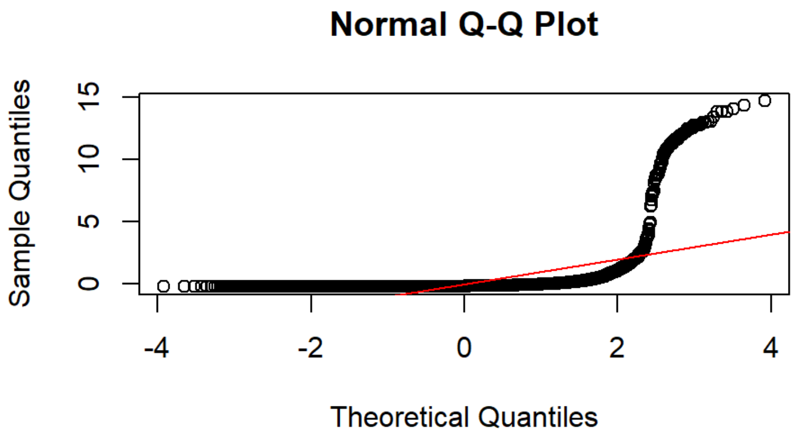

We perform a log-transformation.

```
#log transformation, replace zeros with 1
collapse_rand <- ifelse(as.vector(collapsedAll_random)==0,1,as.vector(collapsedAll_random))
logcollapse_rand <- log(collapse_rand)

collapse_mean2 <- ifelse(as.vector(collapsedAll_mean)==0,1, as.vector(collapsedAll_mean))
logcollapse_mean2 <- log(collapse_mean2)


collapse_median2 <- ifelse(as.vector(collapsedAll_median)==0,1, as.vector(collapsedAll_median))
logcollapse_median2 <- log(collapse_median2)
```

Check exploratory plots again:

```
#RANDOMLY Selected Day
##visual checks and exploration
jj <- scale(as.vector(logcollapse_rand))
qqnorm(jj, main = "QQ-Plot for Log-Transformed Randomly Selected Day")
abline(0, 1, col="red")
```

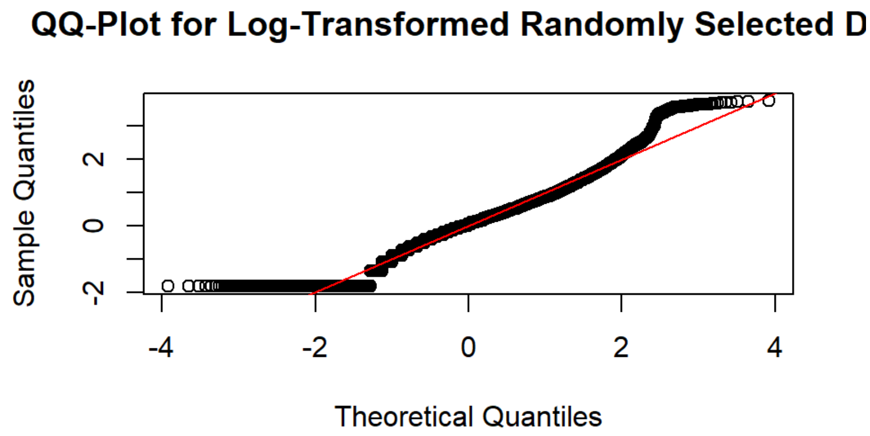


```
plot(density(as.vector(logcollapse_rand)), main="Density Plot for Log-Transformed Randomly Selected Day")
```

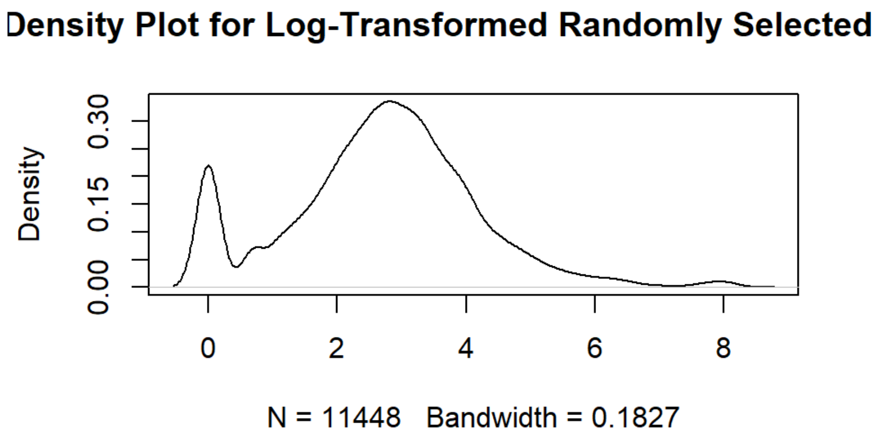


```
#check MEAN
##visual check and exploration
jj <- scale(as.vector(logcollapse_mean2))
qqnorm(jj, main = "QQ-Plot for Log-Transformed Mean")
abline(0, 1, col="red")
```

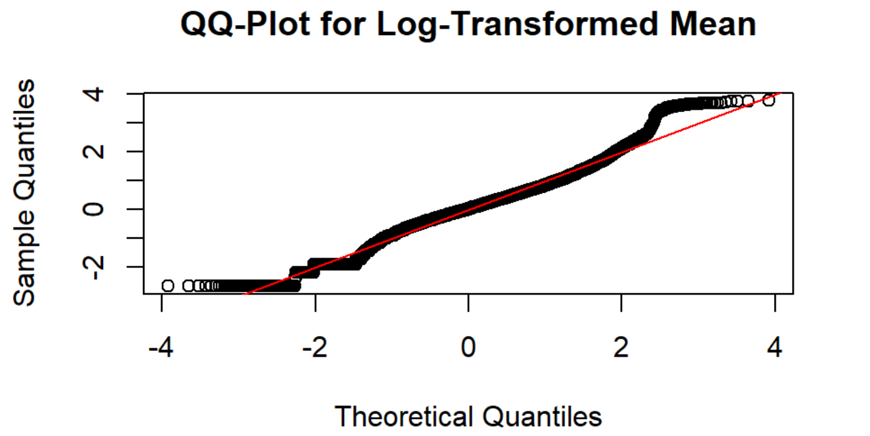

```
plot(density(as.vector(logcollapse_mean2)), main="Density Plot for Log-Transformed Mean")
```

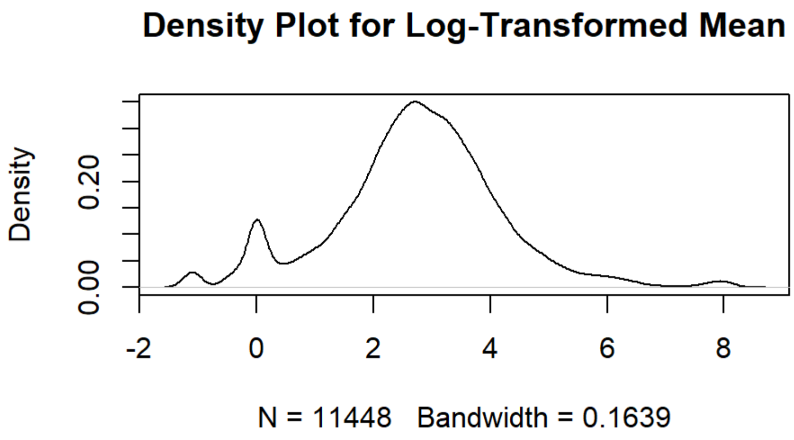


```
#check MEDIAN
##visual check
jj <- scale(as.vector(logcollapse_median2))
qqnorm(jj)
abline(0, 1, col="red", main = "QQ-Plot for Log-Transformed Median")
```

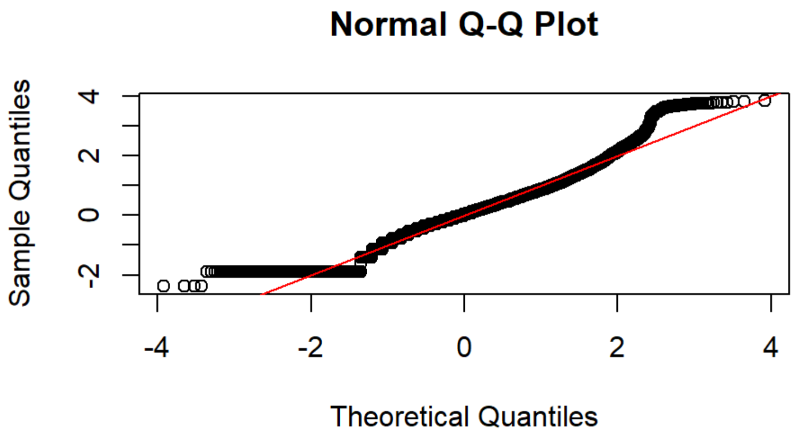


```
plot(density(as.vector(logcollapse_median2)), main="Density Plot for Log-Transformed Randomly Selected Day"
)
```

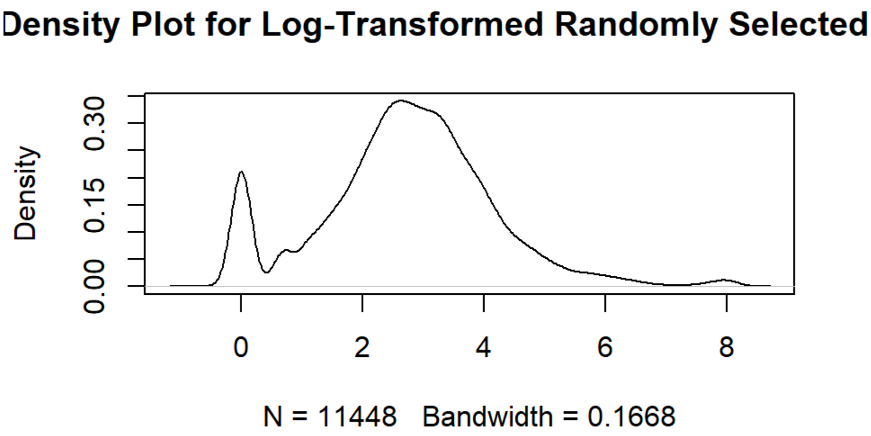

Performing TOST and RTOST:

```
#TOSTS
#Random vs Mean
d <- tost(logcollapse_rand, logcollapse_mean2, paired=TRUE, conf.level=0.95, epsilon=0.25,tost.p.value=0.05
, var.equal = TRUE)
cbind(round(d$tost.p.value,3), d$result)
```

```
##      [,1] [,2]
## [1,] "0"  "rejected"
```

```
#Random vs Median
e <- tost(logcollapse_rand, logcollapse_median2, paired=TRUE, conf.level=0.95, epsilon=0.25,tost.p.value=0.
05, var.equal=TRUE)
cbind(round(e$tost.p.value,3), e$result)
```

```
##      [,1] [,2]
## [1,] "0"  "rejected"
```

```
##RTOSTS
# #Random vs Mean
robust_2 <- rtost(logcollapse_rand, logcollapse_mean2, alpha=0.05, epsilon=0.25, tr=0.2,paired=TRUE)
cbind(round(robust_2$p.value,2), robust_2$result)
```

```
##      [,1] [,2]
## [1,] "0"  "rejected"
```

```
# #Random vs Median
robust_1 <- rtost(logcollapse_rand, logcollapse_median2, alpha=0.05, epsilon=0.25, tr=0.2,paired=TRUE)
cbind(round(robust_1$p.value,2), robust_1$result)
```

```
##      [,1] [,2]
## [1,] "0"  "rejected"
```

NMDS Plotting

```
library(vegan)
library(ggplot2)
```

```
#Using first lactation data normalized to 9,100 sequences with the 0.1% abundance cutoff applied

#Load and order the OTU table and meta file. Adding some more columns to make subsetting easier
meta <- read.table("FirstYearMeta.txt", header=TRUE, row.names=1, sep="\t")
meta <- meta[order(row.names(meta)),]
meta$period.day <- paste(meta$lact.period, meta$lact.day)
meta$animal.period <- paste(meta$cow, meta$lact.period)
meta$grp <- paste(meta$cow,meta$type, meta$lact.period)
OTUTable <- read.csv("FirstYearShared.txt", header=TRUE, row.names=1, sep="\t")
OTUTable <- OTUTable[order(row.names(OTUTable)),]

meta$pch <- ifelse(meta$lact.period=="early", 15, ifelse(meta$lact.period=="middle", 16, 17))

meta$type.period <- paste(c=meta$type, meta$lact.period)
meta$type.period.pch <- ifelse(meta$type.period=="liquid early", 0, ifelse(meta$type.period=="liquid middl
e", 1, ifelse(meta$type.period=="liquid late", 2, ifelse(meta$type.period=="solid early", 15, ifelse(meta$t
ype.period=="solid middle", 16, 17)))))

meta$col <- ifelse(meta$cow=="4255","#a6cee3",ifelse(meta$cow=="4260", "#1f78b4",ifelse(meta$cow=="4261","#
b2df8a",ifelse(meta$cow=="4262","#33a02c",ifelse(meta$cow=="4273","#fb9a99",ifelse(meta$cow=="4276","#e31a1
c",ifelse(meta$cow=="4277","#fdbf6f",ifelse(meta$cow=="4278","#ff7f00",ifelse(meta$cow=="4281","#cab2d6", i
felse(meta$cow=="4282","#6a3d9a",ifelse(meta$cow=="4294","#ffff99","#b15928")))))))))))

#Subsetting the meta and OTU tables
meta.solid <- meta[which(meta$type=="solid"),]
OTUTable.solid <- OTUTable[which(meta$type=="solid"),]
meta.liquid <- meta[which(meta$type=="liquid"),]
OTUTable.liquid <- OTUTable[which(meta$type=="liquid"),]
```

```
#Measuring and displaying beta diversity for Solid first lactation samples.
OTU.dist=vegdist(OTUTable, distance="bray")

adonis(OTUTable ~ lact.day, data = meta, permutations = 1000)
```

```
##
## Call:
## adonis(formula = OTUTable ~ lact.day, data = meta, permutations = 1000)
##
## Permutation: free
## Number of permutations: 1000
##
## Terms added sequentially (first to last)
##
##            Df SumsOfSqs  MeanSqs F.Model      R2 Pr(>F)
## lact.day    1    0.0087 0.008675 0.15697 0.00074  0.998
## Residuals 213   11.7714 0.055265         0.99926
## Total     214   11.7801                  1.00000
```

```
bdisp <- betadisper(OTU.dist, meta$cow)
permutest(bdisp, pairwise=TRUE, permutations=500)
```

```
##
## Permutation test for homogeneity of multivariate dispersions
## Permutation: free
## Number of permutations: 500
##
## Response: Distances
##            Df  Sum Sq   Mean Sq      F N.Perm   Pr(>F)
## Groups     11 0.17953 0.0163208 9.3441    500 0.001996 **
## Residuals 203 0.35457 0.0017466
## ---
## Signif. codes:  0 '***' 0.001 '**' 0.01 '*' 0.05 '.' 0.1 ' ' 1
##
## Pairwise comparisons:
## (Observed p-value below diagonal, permuted p-value above diagonal)
##            4255       4260       4261       4262       4273       4276
## 4255            1.9960e-03 3.9920e-03 3.9920e-03 3.9920e-03 1.9960e-03
## 4260 3.4638e-05            3.6128e-01 1.9960e-03 9.2415e-01 5.6088e-01
## 4261 4.2587e-03 3.2543e-01            1.9960e-03 4.7505e-01 5.8483e-01
## 4262 1.1550e-03 2.0273e-08 8.5760e-06            1.9960e-03 1.9960e-03
## 4273 2.9745e-03 9.1804e-01 4.9460e-01 2.5702e-05            7.7046e-01
## 4276 3.3915e-05 5.7721e-01 5.7717e-01 5.4863e-09 7.6122e-01
## 4277 7.4144e-02 1.0767e-06 2.0136e-04 1.8840e-01 2.5819e-04 6.9372e-07
## 4278 2.8407e-01 6.5816e-03 9.7349e-02 1.6883e-03 3.9923e-02 1.3509e-02
## 4281 2.7322e-01 1.2188e-02 1.3293e-01 2.6334e-03 5.4309e-02 2.5114e-02
## 4282 1.6983e-01 4.0327e-03 8.8207e-02 1.8300e-04 3.8106e-02 7.6400e-03
## 4294 9.2217e-01 2.9513e-05 4.2325e-03 4.5548e-04 3.0308e-03 2.6306e-05
## 4297 6.2573e-02 1.7999e-06 2.1435e-04 4.0766e-01 2.4078e-04 1.5327e-06
##            4277       4278       4281       4282       4294   4297
## 4255 7.9840e-02 2.3553e-01 2.6747e-01 1.8164e-01 9.3014e-01 0.0679
## 4260 1.9960e-03 1.9960e-03 3.9920e-03 1.3972e-02 1.9960e-03 0.0020
## 4261 1.9960e-03 1.0978e-01 1.4970e-01 9.3812e-02 5.9880e-03 0.0020
## 4262 2.0160e-01 3.9920e-03 1.9960e-03 1.9960e-03 1.9960e-03 0.4112
## 4273 1.9960e-03 3.9920e-02 6.9860e-02 5.7884e-02 5.9880e-03 0.0020
## 4276 1.9960e-03 7.9840e-03 1.9960e-02 1.1976e-02 1.9960e-03 0.0020
## 4277            1.1976e-02 2.3952e-02 7.9840e-03 4.5908e-02 0.7725
## 4278 2.5636e-02            9.1018e-01 9.4212e-01 3.0938e-01 0.0160
## 4281 2.9659e-02 9.2871e-01            9.9002e-01 3.1138e-01 0.0140
## 4282 7.8098e-03 9.3205e-01 9.8669e-01            1.6567e-01 0.0040
## 4294 5.1096e-02 3.0102e-01 2.8897e-01 1.7776e-01            0.0439
## 4297 7.6936e-01 2.1476e-02 2.4427e-02 7.7324e-03 4.5677e-02
```

```
#Plotting the Solid Fraction
bray.ord.s <- metaMDS(OTUTable.solid, distance="bray", trymax = 1000)
```

```
## Square root transformation
## Wisconsin double standardization
## Run 0 stress 0.09288861
## Run 1 stress 0.1160054
## Run 2 stress 0.09288873
## ... Procrustes: rmse 2.813539e-05  max resid 0.0001717143
## ... Similar to previous best
## Run 3 stress 0.09288868
## ... Procrustes: rmse 5.315399e-05  max resid 0.0004289785
## ... Similar to previous best
## Run 4 stress 0.09288895
## ... Procrustes: rmse 7.769554e-05  max resid 0.0005845752
## ... Similar to previous best
## Run 5 stress 0.1120599
## Run 6 stress 0.09288891
## ... Procrustes: rmse 5.242478e-05  max resid 0.000382655
## ... Similar to previous best
## Run 7 stress 0.09288866
## ... Procrustes: rmse 6.012411e-05  max resid 0.0004115171
## ... Similar to previous best
## Run 8 stress 0.09288863
## ... Procrustes: rmse 3.489897e-05  max resid 0.0003030999
## ... Similar to previous best
## Run 9 stress 0.09288862
## ... Procrustes: rmse 2.512154e-05  max resid 0.0001917786
## ... Similar to previous best
## Run 10 stress 0.09288866
## ... Procrustes: rmse 5.31791e-05  max resid 0.000385181
## ... Similar to previous best
## Run 11 stress 0.0928888
## ... Procrustes: rmse 7.957117e-05  max resid 0.0006020443
## ... Similar to previous best
## Run 12 stress 0.1120596
## Run 13 stress 0.1224345
## Run 14 stress 0.09288863
## ... Procrustes: rmse 2.609086e-05  max resid 0.0001990263
## ... Similar to previous best
## Run 15 stress 0.09288863
## ... Procrustes: rmse 4.463609e-05  max resid 0.0002996866
## ... Similar to previous best
## Run 16 stress 0.1160431
## Run 17 stress 0.09288862
## ... Procrustes: rmse 3.678582e-05  max resid 0.0002321787
## ... Similar to previous best
## Run 18 stress 0.1120596
## Run 19 stress 0.0928886
## ... New best solution
## ... Procrustes: rmse 2.102164e-05  max resid 0.0001577386
## ... Similar to previous best
## Run 20 stress 0.09288865
## ... Procrustes: rmse 3.762214e-05  max resid 0.0002938863
## ... Similar to previous best
## *** Solution reached
```

```
plot.new()
plot(bray.ord.s, type="n", display="sites", main="All Cows Solid Fraction")
points(bray.ord.s, display = "sites", pch = meta.solid$pch, col = meta.solid$col)
legend("topright", col=c("#a6cee3", "#1f78b4", "#b2df8a", "#33a02c", "#fb9a99", "#e31a1c", "#fdbf6f", "#ff7
f00", "#cab2d6", "#6a3d9a", "#ffff99", "#b15928"), legend=c("4255", "4260", "4261", "4262", "4273", "4276",
 "4277", "4278", "4281", "4282", "4294", "4297"), pch=15, cex=0.7)
legend("bottomright", legend=c("Early", "Middle", "Late"), col="black", pch=c(15, 16, 17))
```

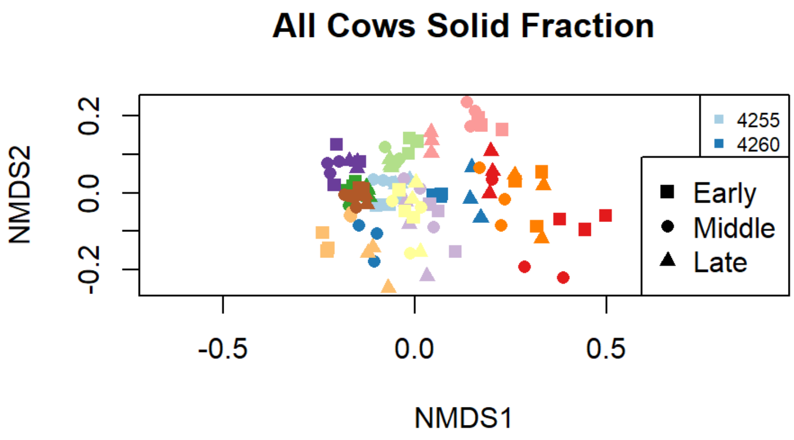


```
#ordiellipse(bray.ord.s, groups = meta.solid$animal.period,  draw="lines", kind="se", label=TRUE, display
="sites")

#Plotting the Liquid Fraction
bray.ord.l <- metaMDS(OTUTable.liquid, distance="bray", trymax = 1000)
```

```
## Square root transformation
## Wisconsin double standardization
## Run 0 stress 0.1192918
## Run 1 stress 0.1429527
## Run 2 stress 0.1463814
## Run 3 stress 0.1345937
## Run 4 stress 0.1504765
## Run 5 stress 0.1349794
## Run 6 stress 0.1350053
## Run 7 stress 0.1568562
## Run 8 stress 0.1550491
## Run 9 stress 0.1192613
## ... New best solution
## ... Procrustes: rmse 0.0009821212  max resid 0.00842971
## ... Similar to previous best
## Run 10 stress 0.1571213
## Run 11 stress 0.1206109
## Run 12 stress 0.146187
## Run 13 stress 0.1666493
## Run 14 stress 0.1437293
## Run 15 stress 0.1490113
## Run 16 stress 0.1422375
## Run 17 stress 0.1192534
## ... New best solution
## ... Procrustes: rmse 0.001905247  max resid 0.01058145
## Run 18 stress 0.1192449
## ... New best solution
## ... Procrustes: rmse 0.001652242  max resid 0.01051453
## Run 19 stress 0.1314624
## Run 20 stress 0.134579
## Run 21 stress 0.1498949
## Run 22 stress 0.13563
## Run 23 stress 0.1192631
## ... Procrustes: rmse 0.001019492  max resid 0.007088089
## ... Similar to previous best
## *** Solution reached
```

```
plot.new()
plot(bray.ord.l, type="n", display="sites", main="All Cows Liquid Fraction")
legend("topright", col=c("#a6cee3", "#1f78b4", "#b2df8a", "#33a02c", "#fb9a99", "#e31a1c", "#fdbf6f", "#ff7
f00", "#cab2d6", "#6a3d9a", "#ffff99", "#b15928"), legend=c("4255", "4260", "4261", "4262", "4273", "4276",
 "4277", "4278", "4281", "4282", "4294", "4297"), pch=15, cex=0.7)
legend("bottomright", legend=c("Early", "Middle", "Late"), col="black", pch=c(15, 16, 17))
points(bray.ord.l, display = "sites", pch=meta.liquid$pch, col = meta.liquid$col)
```

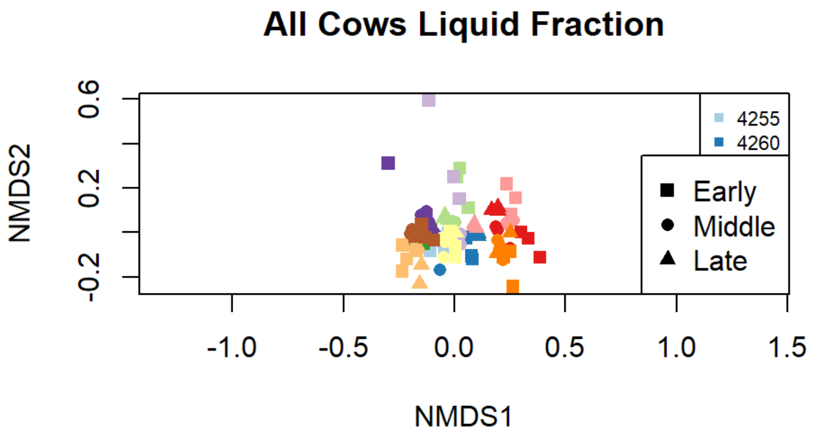


```
#ordiellipse(bray.ord.l, groups = meta.liquid$animal.period,  draw="lines", kind="se", label=TRUE, display
="sites")

#Plotting Both Fractions Together
bray.ord.all <- metaMDS(OTUTable, distance="bray", trymax=1000)
```

```
## Square root transformation
## Wisconsin double standardization
## Run 0 stress 0.1305441
## Run 1 stress 0.1305414
## ... New best solution
## ... Procrustes: rmse 0.0007451393  max resid 0.009815833
## ... Similar to previous best
## Run 2 stress 0.1458389
## Run 3 stress 0.1305445
## ... Procrustes: rmse 0.0002220454  max resid 0.003141466
## ... Similar to previous best
## Run 4 stress 0.1305464
## ... Procrustes: rmse 0.0003186776  max resid 0.004507646
## ... Similar to previous best
## Run 5 stress 0.1305399
## ... New best solution
## ... Procrustes: rmse 0.0003395803  max resid 0.004813939
## ... Similar to previous best
## Run 6 stress 0.1346677
## Run 7 stress 0.13054
## ... Procrustes: rmse 0.0001435083  max resid 0.002053804
## ... Similar to previous best
## Run 8 stress 0.1898705
## Run 9 stress 0.1346602
## Run 10 stress 0.1305461
## ... Procrustes: rmse 0.0006447662  max resid 0.009132202
## ... Similar to previous best
## Run 11 stress 0.1346673
## Run 12 stress 0.1463567
## Run 13 stress 0.1305419
## ... Procrustes: rmse 0.0003166963  max resid 0.004470312
## ... Similar to previous best
## Run 14 stress 0.1977914
## Run 15 stress 0.1346579
## Run 16 stress 0.1305399
## ... New best solution
## ... Procrustes: rmse 1.485356e-05  max resid 0.0001988255
## ... Similar to previous best
## Run 17 stress 0.1305412
## ... Procrustes: rmse 0.0002688537  max resid 0.003267448
## ... Similar to previous best
## Run 18 stress 0.1305405
## ... Procrustes: rmse 0.000217128  max resid 0.003103176
## ... Similar to previous best
## Run 19 stress 0.1346648
## Run 20 stress 0.1305435
## ... Procrustes: rmse 0.0004151445  max resid 0.00585213
## ... Similar to previous best
## *** Solution reached
```

```
plot.new()
plot(bray.ord.all, type="n", display="sites", main="All Cows Both Fractions")
points(bray.ord.all, display="sites", pch=meta$type.period.pch, col=meta$col)
legend("topright", col=c("#a6cee3", "#1f78b4", "#b2df8a", "#33a02c", "#fb9a99", "#e31a1c", "#fdbf6f", "#ff7
f00", "#cab2d6", "#6a3d9a", "#ffff99", "#b15928"), legend=c("4255", "4260", "4261", "4262", "4273", "4276",
 "4277", "4278", "4281", "4282", "4294", "4297"), pch=15, cex=0.7)
legend("bottomright", legend=c("Liquid Early", "Liquid Middle", "Liquid Late", "Solid Early", "Solid Middl
e", "Solid Late"), col="black", pch=c(0, 1, 2, 15, 16, 17))
```

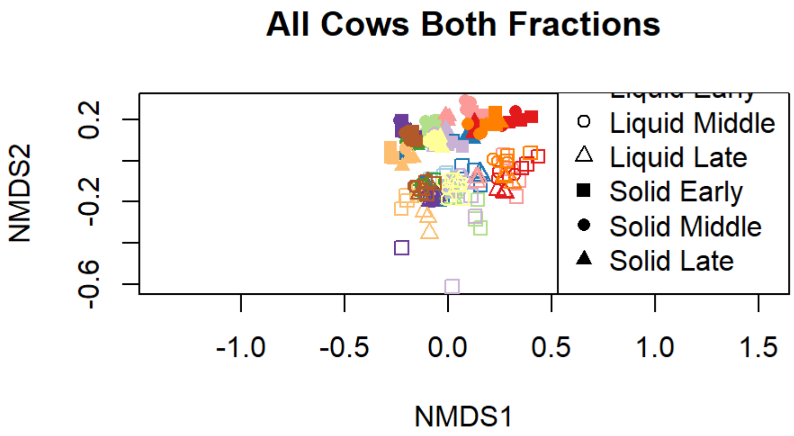
All Cows Both Fractions
NMDS2
0.2
-0.2
-0.6
-1.0
-0.5
0.0
0.5
1.0
1.5
NMDS1
Liquid Middle
Liquid Late
Solid Early
Solid Middle
Solid Late
