# Supplementary material for: The ruminal bacterial community in lactating dairy cows has limited variation on a day-to-day basis
Source: J Anim Sci Biotechnol. 2019 Aug 19;10:66. doi: 10.1186/s40104-019-0375-0 (PMC6698983; doi:10.1186/s40104-019-0375-0)
Supplement: Supplementary file 3 — Figures S1 and S2. Bray-Curtis NMDS Plots of the Ruminal Solid and Liquid Fractions. (DOCX 28 kb) [file 40104_2019_375_MOESM3_ESM.docx]

**Supplementary Figures**

**
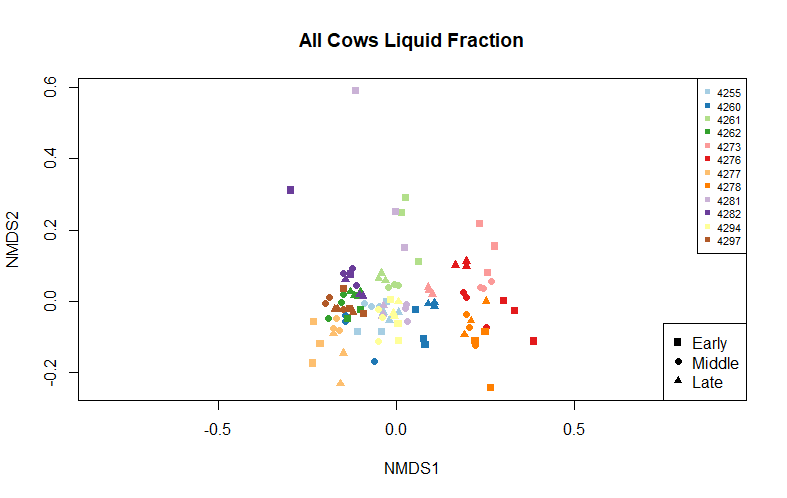
Figure S1.** Non-metric multidimensional scaling (NMDS) plot of the liquid fraction samples. Distances were calculated using the Bray-Curtis dissimilarity index in the *vegan* package in R. Points were plotted based on subject and period of lactation.


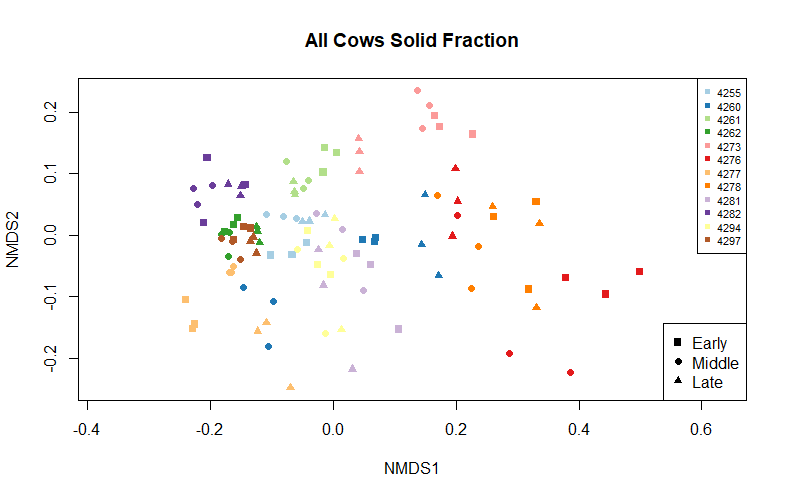


**Figure S2.** NMDS plot of the solid fraction samples. Distances were calculated using the Bray-Curtis dissimilarity index in the *vegan* package in R. Points were plotted based on subject and period of lactation.
